# enDigital Postpartum Support for Early Risk Identification Among Postpartum Women: Formative Randomized Evaluation and Exploratory Predictive Modeling Study

**DOI:** 10.2196/89719

**Published:** 2026-07-15

**Authors:** Lisa Marceau, James Matuk, Jennifer Barkin, Diana Pfeil, Ariana Buterbaugh, Danielle C Perry, Madison Soucie, Allison Bryant

**Affiliations:** 1 Joyuus, LLC, 1800 Mendon Road, E302, Cumberland, RI, 02864, United States, 1 4014284197; 2 Department of Epidemiology, University of Pittsburgh, Pittsburgh, PA, United States; 3 Department of Health Education and Promotion, East Carolina University, Greenville, NC, United States; 4 Joy, LLC, Boulder, CO, United States; 5 Brown University, Providence, RI, United States; 6 Department of Obstetrics and Gynecology, Mass General Brigham, Boston, MA, United States

**Keywords:** postpartum depression, maternal functioning, SaaS, digital health, usability, predictive data, sofware as a service

## Abstract

**Background:**

The postpartum period represents a critical window for maternal health, yet many individuals lack sustained support and timely identification of physical and mental health risks. Digital health interventions offer a scalable approach to extend care beyond clinical settings. Yet, key elements, including real-world challenges, usability, and effectiveness of such platforms, are insufficiently characterized in this literature.

**Objective:**

This study aimed to conduct a formative randomized evaluation to assess the feasibility, engagement, and preliminary signals of impact of the Joyuus platform and to examine the potential for early identification of postpartum health risks.

**Methods:**

We conducted a 12-week randomized evaluation with postpartum participants recruited through community-based organizations. Participants were randomized to either the Joyuus intervention or standard postpartum care. Primary and secondary outcomes included the Barkin Index of Maternal Functioning, the Edinburgh Postnatal Depression Scale (EPDS), the State-Trait Anxiety Inventory, and the Connor-Davidson Resilience Scale. Analyses were conducted using an intention-to-treat approach with linear regression models adjusting for baseline values. Engagement metrics (ie, sessions, time on site, and feature use) were captured through in-app analytics. An exploratory predictive model was developed using baseline clinical, behavioral, and demographic variables to identify individuals at risk for postpartum depression.

**Results:**

Baseline characteristics were generally balanced across arms, and no statistically significant differences were observed in education, income, or marital status. No statistically significant differences were found between treatment and control groups in the 12-week changes for the primary or secondary outcomes. Mean EPDS scores were 9.7 in the intervention group and 9.3 in the control group. In the sample, 60 (45.5%) of the 132 participants met the criteria for elevated depression (EPDS score ≥11 or a positive response to question 10 on self-harm), indicating a high burden of symptoms within the study population. Engagement with the platform was highest during the first 4 weeks. Among intervention participants, 80% created an account. Participants reported high levels of perceived usefulness, ease of use, and relevance of content. Qualitative analysis of open-ended survey responses highlighted limited awareness of postpartum-specific resources and a preference for simple, accessible information. An exploratory predictive model demonstrated a recall of 0.89 and a precision of 0.73 in identifying individuals at risk for postpartum depression, suggesting the feasibility of early risk identification using integrated data inputs.

**Conclusions:**

Joyuus demonstrated feasibility and acceptability but did not produce statistically significant improvements in maternal functioning or maternal health outcomes over 12 weeks. Joyuus identified high rates of depressive symptoms and early engagement patterns, which suggest an opportunity for earlier identification of risk and intervention during the postpartum period. Exploratory modeling results indicate the potential for data-driven approaches to support earlier detection. Future work includes optimizing engagement strategies and expanding validation of predictive detection to improve postpartum surveillance and outcomes.

## Introduction

### Background

The postpartum period is one of the most vulnerable times for maternal health, yet it remains significantly underserved by the health care system, particularly for individuals facing socioeconomic disadvantage, structural barriers to care, and differential access to postpartum support services [1-4]. These disparities are shaped by intersecting psychosocial and structural determinants, including access to care, insurance continuity, social support, employment changes, and environmental stressors. While racial and ethnic disparities in maternal outcomes are well documented, these differences reflect broader structural and social determinants that influence access to care and continuity of coverage. Our study focuses on the broader set of clinical, behavioral, and social determinants influencing postpartum health outcomes.

The United States has the highest maternal mortality rate among high-income nations, driven by systemic health disparities [5], yet research shows that nearly 3 in 4 maternal deaths are preventable [6]. Social determinants of health (ie, external health factors), including income, education, employment, social support, housing stability, and access to health care, all play a critical role in shaping maternal and postpartum outcomes. A substantial share of serious maternal morbidity and mortality occurs after discharge and well past the early postpartum weeks, underscoring the need for surveillance and support beyond the first few months [7,8].

Clinical challenges during this period include persistent hypertension, cardiomyopathy, infection, pain syndromes, pelvic floor dysfunction, and lactation complications, alongside mental health conditions such as depression and anxiety that often emerge or intensify after birth. Current American College of Obstetricians and Gynecologists guidelines recommend treating postpartum care as an ongoing process rather than a single encounter, assessing physical, mental, and social well-being [9]. Clinical care alone is insufficient without attention to real-world pressures that shape health after birth. Return-to-work timelines, availability of paid leave, job security, childcare access, housing stability, and food and financial insecurity all influence maternal mental and physical outcomes. Further, considerations related to health care service use and access to recommended care influence these outcomes. Structural drivers of outcomes also interact with inequities by race, ethnicity, and income, which disproportionately face the steepest barriers to diagnosis, treatment, and recovery [10,11].

Digital health products and interventions have expanded in the postpartum space, promising scalable support for screening, symptom tracking, education, lactation help, and remote monitoring [12]. Randomized and quasi-experimental studies suggest that digital health interventions can produce modest yet meaningful reductions in postpartum depression (PPD) and anxiety symptoms, and emerging work in remote cardiovascular monitoring shows feasibility for detecting and managing risk [13]. These important tools are often highly targeted, focusing on mood screening, pelvic floor exercise, blood pressure checks, or breastfeeding rather than providing a coordinated platform that integrates medical, mental, and social determinants across the full year. As a result, women and health care providers are faced with navigating multiple disconnected apps and portals, which can exacerbate underdiagnosis and misdiagnosis. Symptoms of postpartum medical conditions can be nonspecific (eg, fatigue, dyspnea, and pain) and overlap with expected recovery, while stigma, logistical barriers, and limited postpartum contact reduce opportunities for detection. Even when screening is recommended, follow-up and referral pathways are not consistently implemented, particularly in settings serving high-risk populations with substantial unmet social needs. These gaps contribute to avoidable complications and missed chances to intervene early when outcomes can be shifted [6].

### PPD Risk and Underdiagnosis

Mental health conditions, such as PPD, anxiety, and substance use disorders, are leading causes of maternal death, accounting for over 20% of fatalities during the postpartum period [14]. PPD is one of the most common complications after childbirth and is linked to impaired maternal functioning, reduced engagement with health care services, difficulties with lactation and bonding, and adverse child socioemotional and developmental outcomes [15]. The reported global prevalence of PPD is estimated at 17.22% [16] and is estimated at 13% in the US population [17], although these estimates vary by demographics and are considered to underreport the true prevalence. Mental health challenges are often combined with or exacerbated by postpartum physical health complications [18]. Conditions such as postpartum hemorrhage, hypertension, infections, and cardiac complications are leading causes of maternal mortality alongside mental health [19,20].

Both physical and mental health conditions are exacerbated by factors such as inadequate health care access, unstable living conditions, lack of health insurance, and environmental factors such as heat risk and exposure to extreme weather [21,22]. As a result, individuals are less likely to attend postpartum checkups, receive appropriate follow-up care, or be monitored for complications such as high blood pressure or infection. Health care coverage is a crucial external health factor impacting postpartum outcomes [23]. Gaps in insurance coverage contribute to missed opportunities for preventive care and follow-up. Medicaid, which covers approximately 42% of US births, still fails to meet postpartum needs. While the American Rescue Plan Act of 2021 [24] provided the option to extend coverage from 60 days to 12 months, gaps remain in many states, leaving individuals uninsured during a critical period for health monitoring [23]. Those in rural or underserved urban maternal care deserts face additional challenges, where access to maternal health care services is limited or nonexistent [25]. The postpartum coverage patchwork puts individuals at risk by requiring them to determine when they need care, often when it becomes urgent, during an already overwhelming period.

### Point Solutions to Integrated Platform

The evidence that digital health interventions can improve health outcomes is encouraging, but real-world impact will depend on integration [26-28]. Addressing postpartum health requires the full spectrum of clinical and nonclinical factors that contribute to or exacerbate health challenges. Joyuus is a software-as-a-service platform that addresses postpartum self-care through a comprehensive, evidence-based platform integrating mental, physical, social, and real-world concerns across the first year postpartum. The Joyuus platform includes evidence-based content across mental, physical, social, and real-world postpartum domains, including validated screening tools, red flag alerts, which indicate clinical risk, and resource navigation to connect users to resources and providers. Users access the content through a mobile- or web-based interface with audio, bookmark, and relevance features to address time constraints. As a health care integrator, Joyuus aggregates information, identifies risk, and connects users to care providers through safety-net organizations, employer benefits, and payors. Beyond identifying clinical and mental health risk factors, Joyuus has created a comprehensive resource covering a range of topics that address the main question concerning individuals postpartum: “Am I okay?” This can range from questions such as “Why do my feet grow?” or “How do I manage pets with a newborn?” to understanding risks for depression, hypertension, food insecurity, and other aspects of postpartum that may require intervention. The purpose of this study was to conduct a formative randomized evaluation to assess the feasibility, engagement, and preliminary signals of impact of the Joyuus platform and to examine the potential for early identification of postpartum health risks.

## Methods

### Sample Size and Power

This study was a 2-arm randomized controlled trial designed to evaluate the Joyuus platform, including baseline, 6-week, and 12-week data collection timepoints. Outcomes included maternal functioning, depression, anxiety, social support, resilience, and knowledge, detailed below. The trial was registered at ClinicalTrials.gov (NCT05876559).

Participants were recruited from community-based postpartum organizations nationally. Recruitment was conducted through a tiered process, including outreach and initial screening to identify bots or survey scam companies attempting to access the survey platform. Once a potential participant was identified by a community organization or research staff, the study screening, consent, and survey process were conducted through Qualtrics (Qualtrics, LLC), a Health Insurance Portability and Accountability Act–compliant, Health Information Trust Alliance–certified survey platform. Qualtrics also performs several checks to flag inappropriate access, which were also validated. Participants were eligible for the study if they were a new mother (≤6 months postpartum), had experienced a live birth, and were aged 18 years or older. Participants had to be able to read, write, and speak English, as all research and product development activities were conducted in English at this stage. Research activities were conducted via smartphone with internet access, which was also a requirement of participation. The goal of Joyuus is wide accessibility; therefore, no restrictions were placed on phone model, type of Wi-Fi, bandwidth, or where participants accessed Joyuus. Participants who reported Edinburgh Postnatal Depression Scale (EPDS) summary scores of >11, or any positive response on question 10 regarding self-harm, were considered at risk for depression and were reviewed by the Safety Monitoring Committee.

Participants were recruited using a multipronged approach, including collaboration with health care providers, partnership with organizations focused on supporting individuals during postpartum, and through advertising on private Facebook (Meta Platforms, Inc) groups specifically for new mothers.

The trial was designed to enroll 132 participants randomized in a 1:1 allocation ratio. This target sample size was determined to detect a difference in 12-week change in the Barkin Index of Maternal Function (BIMF) of 7.96 (SD 16.2), corresponding to a standardized effect size of 0.49, with 80% power.

Primary analyses were conducted using the intention-to-treat principle, including all randomized participants according to assigned groups. Sensitivity analyses were conducted to assess the robustness of findings. The expected change in BIMF was based on prior literature and represents a moderate effect size, although a minimal clinically important difference for BIMF has not been definitively established.

### Outcome Measures

#### Overview

The primary outcome was the change in the BIMF from baseline to 12 weeks [29,30]. Secondary outcomes included the EPDS [31], the State-Trait Anxiety Inventory (STAI) [32], the Connor-Davidson Resilience Scale (CD-RISC) [33], the Medical Outcomes Study (MOS) Social Support Survey [34], knowledge, and health care access measures. The BIMF is a 20-item self-report measure evaluating domains relevant to postpartum functioning, including social support, infant care, self-care, mother-child interaction, adjustment, and psychological well-being. The BIMF has demonstrated strong internal consistency, with Cronbach α ranging from 0.83 to 0.87. Depressive symptoms were measured using the EPDS, a widely used and validated instrument with strong reliability and construct validity for identifying postpartum depressive symptoms. Anxiety was assessed using the STAI, which includes separate 20-item subscales for state and trait anxiety and has extensive evidence supporting its reliability and validity. Resilience was measured using the CD-RISC, a 25-item instrument evaluating multiple resilience domains with strong psychometric performance across diverse populations. Knowledge outcomes were assessed using a study-specific multiple-choice instrument developed for this trial to evaluate understanding of mental, physical, social, and real-life postpartum concerns. Baseline demographic and descriptive variables included age, race, ethnicity, parity, weeks postpartum, education, income, employment status, and relationship status. Social support was measured using the MOS Social Support Survey as a descriptive contextual variable, although the intervention was not designed to directly modify social support. Technology preferences, including communication format, frequency, social media use, and digital engagement behaviors, were also collected. Platform engagement metrics included page views, unique visitors, bounce rate, and average daily and weekly session activity.

Qualitative data resulting from open-ended survey questions were analyzed using an inductive, thematic coding approach. A predictive model based on the EPDS outcome was developed to predict depression risk in the study population. Descriptive baseline characteristics and outcome measures are summarized in Table 1 below. We used the student *t* test, Fisher exact test, and the chi-square test as appropriate to assess differences between the control and intervention arms of baseline measures.

**Table 1.** Baseline characteristics of study population by treatment arm. Between-group differences were assessed using the *t* test for age; the Fisher exact test for education, employment, marital status, income, race, ethnicity, Hispanic origin, information about health care, and information about difficult topics; and the chi-square test for region. The table displays summary statistics for baseline characteristics by treatment arm. There do not appear to be large differences in most characteristics between treatment arms, except for self-reported US Census region of residence. In the control arm, there were relatively more participants from the South, whereas the treatment arm included more participants from the Northeast and Midwest. The differences in region between the treatment arms were adjusted for in sensitivity analyses for the primary and secondary end points, and the results were consistent with the main findings.

Baseline characteristic	All participants (n=119)	Control (n=63)	Intervention (n=56)	*P* value
Age (years), mean (SD)	30.5 (4.9)	30.1 (4.8)	30.9 (4.9)	.34
Education, n (%)				.63
Middle, high school, no diploma	8 (6.7)	5 (7.9)	3 (5.4)	
High school graduate or general educational development	22 (18.5)	13 (20.6)	9 (16.1)	
Some college, no degree	20 (16.8)	9 (14.3)	11 (19.6)	
Associate degree: technical or academic	16 (13.4)	7 (11.1)	9 (16.1)	
Bachelor’s degree	23 (19.3)	15 (23.8)	8 (14.3)	
Master’s degree, professional school degree, or doctoral degree	30 (25.2)	14 (22.2)	16 (28.6)	
Employment, n (%)				.19
Actively working	24 (20.2)	16 (25.4)	8 (14.3)	
Temporarily out of workforce	37 (31.1)	15 (23.8)	22 (39.3)	
Out of workforce	56 (47.1)	31 (49.2)	25 (44.6)	
Other	2 (1.7)	1 (1.6)	1 (1.8)	
Relationship status, n (%)				≥.99
Married	67 (56.3)	35 (55.6)	32 (57.1)	
Divorced, widowed, and separated	5 (4.2)	3 (4.8)	2 (3.6)	
Never married	28 (23.5)	15 (23.8)	13 (23.2)	
A member of an unmarried couple	19 (16)	10 (15.9)	9 (16.1)	
Income (US $), n (%)				.99
0-19,999	20 (16.8)	10 (15.9)	10 (17.9)	
20,000-39,999	24 (20.2)	12 (19)	12 (21.4)	
40,000-59,999	25 (21)	14 (22.2)	11 (19.6)	
≥60,000	43 (36.1)	23 (36.5)	20 (35.7)	
Do not know or prefer not to answer	7 (5.9)	4 (6.3)	3 (5.4)	
US Census region, n (%)				.02
Northeast	28 (23.5)	13 (20.6)	15 (26.8)	
South	48 (40.3)	32 (50.8)	16 (28.6)	
Midwest	19 (16)	5 (7.9)	14 (25)	
West	24 (20.2)	13 (20.6)	11 (19.6)	
Race and ethnicity, n (%)				.27
Asian American, American Native, Asian, Native Hawaiian, or Other Pacific Islander	4 (3.4)	2 (3.2)	2 (3.6)	
Black or African American	42 (35.3)	24 (38.1)	18 (32.1)	
White (Hispanic)	7 (5.9)	2 (3.2)	5 (8.9)	
White (non-Hispanic)	48 (40.3)	22 (34.9)	26 (46.4)	
Other	15 (12.6)	11 (17.5)	4 (7.1)	
Prefer not to answer	3 (2.5)	2 (3.2)	1 (1.8)	
Hispanic, n (%)				.43
Yes	17 (14.3)	7 (11.1)	10 (17.9)	
No	102 (85.7)	56 (88.9)	46 (82.1)	
Seeking online information about health care, n (%)				.14
Very frequent	39 (32.8)	22 (34.9)	17 (30.4)	
Somewhat frequent	65 (54.6)	30 (47.6)	35 (62.5)	
Not frequent	15 (12.6)	11 (17.5)	4 (7.1)	
Seeking online information about difficult topics, n (%)				.13
Very frequent	33 (27.7)	21 (33.3)	12 (21.4)	
Somewhat frequent	67 (56.3)	30 (47.6)	37 (66.1)	
Not frequent	19 (16)	12 (19)	7 (12.5)	

#### Analysis for Primary and Secondary End Points

All analyses for primary and secondary end points were conducted using the intention-to-treat principle, whereby participants were analyzed based on the treatment group to which they were randomized, regardless of adherence to their assigned treatment. For the assessment of the 12-week primary end point (BIMF) and most secondary end points (EPDS, STAI, CD-RISC, MOS, knowledge, and health care access), absolute change from baseline was computed by subtracting the measurement at baseline from the 12-week measurement. Linear regression models were used to estimate between-group differences in change scores while adjusting for baseline values. This approach was selected to provide interpretable estimates of treatment effect. In separate linear regression models, the 12-week change was regressed on an intervention group indicator variable and the baseline measurement. The regression coefficient for the intervention group indicator corresponds to the expected difference in 12-week change in the outcome between the treatment and control group. We computed a point estimate for this regression coefficient, along with a 95% CI and a *P* value corresponding to a 2-sided test that the coefficient was equal to 0. We assessed assumptions of the linear model using residual diagnostics. Due to violations of the normality assumption, the trial experience secondary end point, which was collected at the 12-week timepoint, was assessed using the Mann-Whitney *U* test.

We conducted several additional preplanned analyses. We analyzed the 6-week change in primary and secondary end points using the methods described for the 12-week timepoint. We conducted sensitivity analyses that adjusted for baseline covariates believed to have a relationship with primary and secondary end points, as well as covariates that were imbalanced between treatment arms at a .05 significance level. We also considered models with interactions between baseline characteristics and the intervention group indicator variable to assess whether there was treatment effect heterogeneity across participant subgroups. Assessment of primary and secondary end points was conducted using the intention-to-treat principle, whereby participants were grouped for analysis based on the arm to which they were randomly assigned.

We additionally conducted a per-protocol analysis that excluded participants from the intervention arm who did not create an account to use Joyuus. Results from the intention-to-treat and per-protocol analyses were consistent. Within the intervention arm, analytics data were captured that provided information on usage of Joyuus; these data included time spent using the app, sessions on the app, page loads, clicks, searches, and favorites. All hypothesis tests were performed using a significance level of .05. All statistical analyses were conducted using R (version 4.2.3) [35].

Amplitude was used as a data analytics platform, providing comprehensive behavioral insights and user engagement metrics that supported quantitative evaluation of digital platform performance and user interaction trends. Amplitude provides ISO 27001 certification, General Data Protection Regulation and California Consumer Privacy Act support, and secure behavioral analytics data under established privacy and governance frameworks.

### Qualitative Analysis

Baseline qualitative assessment of open-ended survey responses (N=130) was analyzed using an inductive qualitative content analysis approach focused on identifying recurring themes and patterns in participant responses to support quantitative findings. The research team reviewed all responses to identify recurring words, phrases, and concepts. Codes were generated manually and refined through iterative comparison to capture common patterns and divergent perspectives. Responses were then grouped into overarching themes to contextualize quantitative findings and illustrate barriers and opportunities in postpartum digital engagement. This process was descriptive rather than interpretive, focusing on the brief open-ended data, and provided additional insight into participants’ experiences with postpartum digital resources and their communication preferences. The goal of these analyses was not intended to capture complex experiential processes, but rather to provide a large sample size for qualitative data, which resulted in robust descriptive pattern identification.

### Predictive Modeling

#### Overview

An exploratory predictive model was developed to identify postpartum women at elevated risk for depression using baseline data from the Joyuus clinical trial. The model predicts whether participants score above the clinical threshold (EPDS ≥10) for PPD risk. While EPDS is a validated screening tool, the purpose of this exploratory model was to assess whether earlier identification of risk could be achieved using a broader set of clinical, behavioral, and demographic variables before formal screening.

#### Dataset and Variables

The training dataset (N=119) included baseline measurements from seven clinical assessments: BIMF (breastfeeding and maternal functioning), Climate Distress Index, CD-RISC, health care access score, postpartum knowledge score, MOS, and STAI, alongside demographic variables including age, job status, marital status, education level, state of residence, income, race, and ethnicity. Variables were selected based on established literature and empirical associations with PPD.

#### Exploratory Analysis

Before modeling, exploratory data analysis was conducted, including correlation analysis between numeric variables (visualized via a heatmap), univariate distributions (histograms and boxplots), categorical variable frequencies (count plots), and bivariate relationships between predictors and EPDS scores.

#### Model Development

An XGBoost classifier (an implementation of gradient-boosted decision trees) was trained to perform binary classification of depression risk. Categorical variables were one-hot encoded (drop_first=true) to avoid multicollinearity, resulting in an expanded feature set. The data were split 80:20 into training and test sets using stratified sampling to maintain class balance.

Hyperparameter tuning was performed using 3-fold cross-validation grid search with receiver operating characteristic–area under the curve as the evaluation metric. The analysis and model development were done in Python using *pandas* and *NumPy* for data manipulation, and *scikit-learn* and *XGBoost* for training and prediction.

### Ethical Considerations

All research activities, including informed consent, were conducted in accordance with prevailing ethical principles and were reviewed and approved by the WIRB-Copernicus Group Institutional Review Board (OHRP and FDA registration IRB00000533) and an independent Safety Monitoring Committee. Participants were provided compensation of US $50 for baseline survey completion, US $50 for 6-week survey completion, and US $100 for 12-week survey completion. The study was conducted in accordance with the ethical standards laid down in the 1964 Declaration of Helsinki and its later amendments. Participants identified as at risk for depression or self-harm were contacted under a predefined safety monitoring protocol and provided with resources and referral guidance to ensure the ethical management of elevated risk. All participants provided informed consent before study inclusion.

## Results

### Primary and Secondary Overall Outcomes

**Figure 1.**
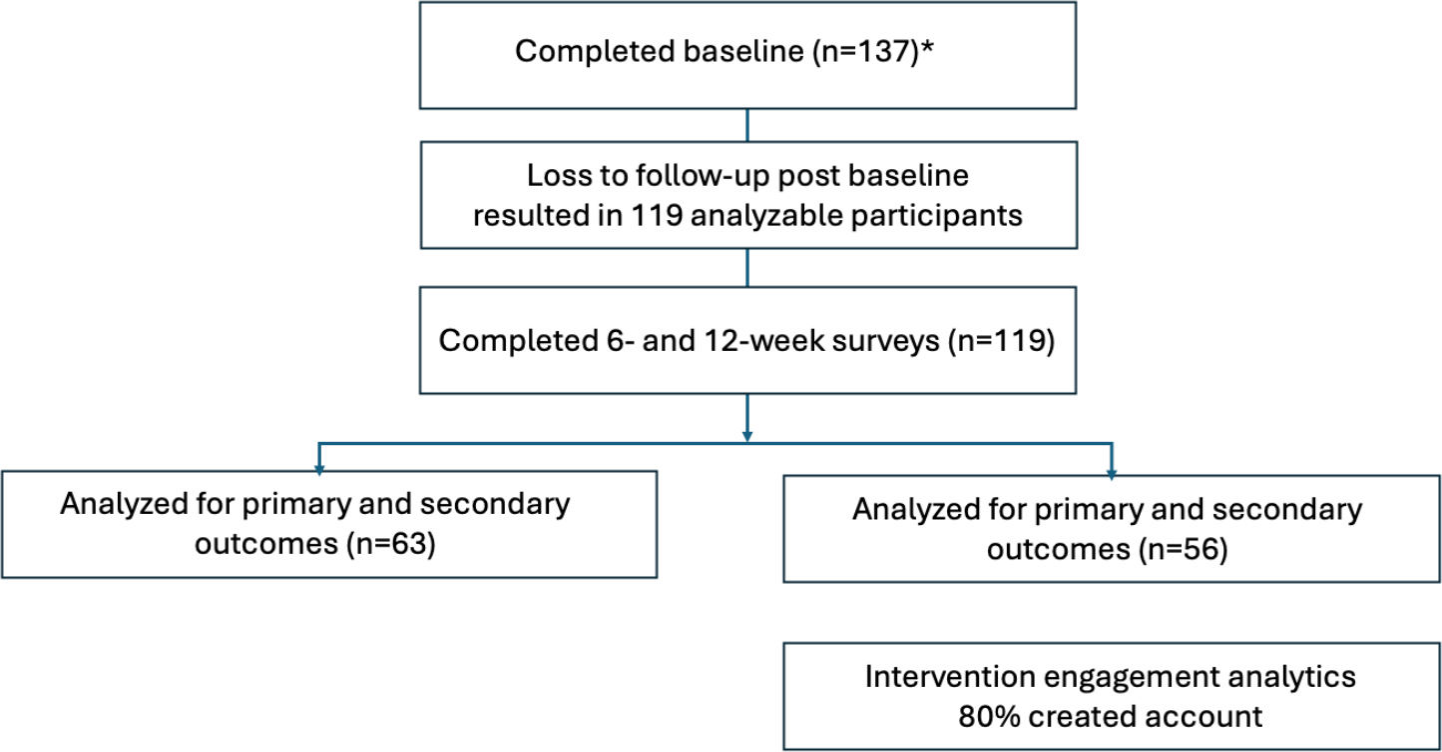
CONSORT (Consolidated Standards of Reporting Trials) flow diagram.

Primary analyses focused on between-group comparisons in change from baseline to 12 weeks. Figure 1 illustrates that although 137 eligible participants completed baseline assessments, outcome data were available for 119 participants at 12 weeks because of attrition, with 63 randomized to the control arm and 56 randomized to the intervention arm. This resulted in an estimated reduction in statistical power to approximately 74%, which increased the risk of type II error, and the final sample imbalance is a potential source of bias. There were no statistically significant differences between intervention and control baseline characteristics. Baseline demographics, described in Table 1, were generally balanced across arms (mean age 30.5 years, SD 4.9 years). No significant differences were observed in education, income, or marital status. There do not appear to be large differences in most characteristics between treatment arms, except for self-reported US Census region of residence. In the control arm, there are relatively more participants from the South, while the treatment arm has more participants from the Northeast and Midwest. The differences in regionality between the arms were adjusted for in sensitivity analyses for the primary and secondary end points, which produced results that were consistent with the main results. The study sample reflects the diversity of the US postpartum population, with representation across income, education, employment, geographic region, race, and ethnicity. While participation required digital access, this aligns with how postpartum individuals seek information and support.

No significant differences were found between treatment and control groups in baseline measurements for the primary (BIMF) or secondary outcomes (EPDS, STAI, CD-RISC, MOS, knowledge, or health care access), described in Table 2.

**Table 2.** Baseline outcomes by treatment arm. The table presents the mean and SD of the primary and secondary end points measured at baseline. There were no significant differences between the intervention and control arms in baseline outcome measures.

Outcome	All (n=119)	Control (n=63)	Intervention (n=56)	*P* value^a^
BIMF^b^, mean (SD)	88.4 (15.7)	88.1 (15.9)	88.7 (15.7)	.84
EPDS^c^, mean (SD)	9.5 (6.3)	9.7 (6.3)	9.3 (6.3)	.73
STAI^d^, mean (SD)	41.1 (12)	41.5 (12.7)	40.6 (11.3)	.69
^e^CD-RISC, mean (SD)	27.3 (7.9)	27.7 (8.1)	26.9 (7.7)	.60
MOS^f^, mean (SD)	67.5 (19.6)	67.9 (19.1)	67 (20.3)	.79
Knowledge, mean (SD)	60 (11.8)	59.4 (12.6)	60.8 (10.8)	.50
Health care access, mean (SD)	24.1 (4.6)	24.4 (4.3)	23.8 (4.8)	.45

a Two-sided *t* tests were used to assess the difference in means baseline outcomes between the 2 treatment arms.

b BIMF: Barkin Index of Maternal Function.

c EPDS: Edinburgh Postnatal Depression Scale.

d STAI: State-Trait Anxiety Inventory.

e CD-RISC: Connor-Davidson Resilience Scale.

f MOS: Medical Outcomes Study.

Table 3 demonstrates results from separate linear models used to assess the change in primary and secondary outcomes. All models included a term for treatment arm and adjusted for baseline measurements. Using a significance level of .05 for all outcomes, we did not detect a significant difference in expected 12-week change between treatment and control arms.

**Table 3.** The 12-week change in outcomes by treatment arm. The table summarizes results from separate linear models used to assess primary and secondary outcomes. All models included a term for treatment arm and adjusted for baseline measurements. Using a significance level of .05 for all outcomes, no significant differences in expected 12-week change were detected between the treatment and control arms.

Outcome	All, mean (SD; n=119)	Control, mean (SD; n=63)	Intervention, mean (SD; n=56)	Difference, estimate^a^ (95% CI)	*P* value^b^
BIMF^c^	7.6 (10.8)	9 (11.2)	5.9 (10.1)	−2.9 (−6.61 to 0.82)	.13
EPDS^d^	−2.4 (4.8)	−3.1 (4.5)	−1.5 (4.9)	1.45 (−0.08 to 2.98)	.63
STAI^e^	−3.3 (8.7)	−4.3 (9)	−2.2 (8.3)	1.85 (−1.05 to 4.75)	.21
^f^CD-RISC	1.9 (6)	2.1 (5.9)	1.5 (6.1)	−0.84 (−2.8 to 1.12)	.40
MOS^g^	6.8 (13.1)	8.5 (14.5)	4.9 (11.2)	−3.91 (−8.16 to 0.33)	.07
Knowledge	6.4 (11.4)	7.2 (12.4)	5.5 (10.1)	−0.91 (−4.35 to 2.54)	.60
Health care access	0.9 (4.4)	0.1 (4.6)	1.7 (4)	1.25 (−0.18 to 2.69)	.87

a Difference in expected 12-week change in outcomes estimated from a linear regression model including an indicator variable for the intervention group and adjusting for the baseline outcome.

b *P* value corresponds to a 2-sided test for the difference in expected 12-week change estimated from the linear regression model.

c BIMF: Barkin Index of Maternal Function.

d EPDS: Edinburgh Postnatal Depression Scale.

e STAI: State-Trait Anxiety Inventory.

f CD-RISC: Connor-Davidson Resilience Scale.

g MOS: Medical Outcomes Study.

**Figure 2.**
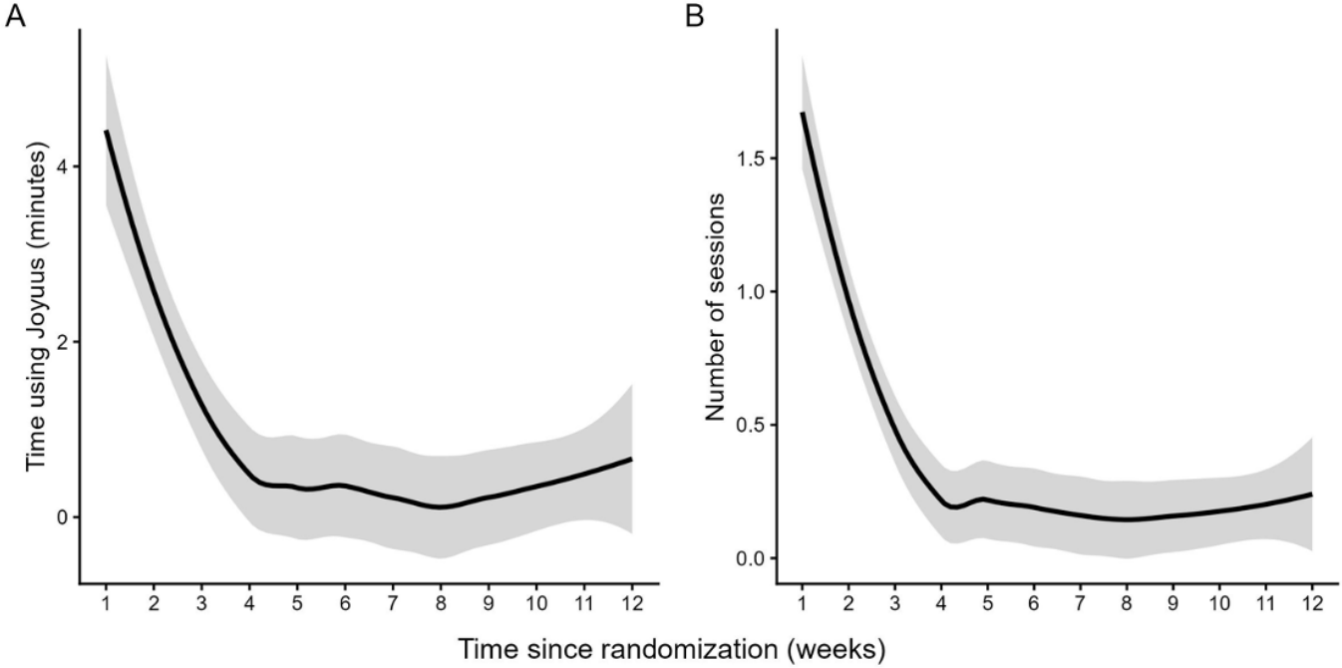
Locally estimated scatterplot smoothing plots of time using Joyuus and the number of sessions within the intervention arm by time since randomization. The figure displays locally estimated scatterplot smoothing plots of time using Joyuus and the number of sessions. The black line displays a moving average of time using Joyuus or the number of sessions throughout the trial, and the gray shading represents a pointwise 95% CI. The plots indicate that most use of the app was concentrated at the beginning of the trial, and use appeared to be negligible on average after about 4 weeks after randomization.

Summary statistics of the trial experience survey and Mann-Whitney *U* test *P* value were used to assess the difference in distributions of the experience survey between treatment and control arms. Intervention participants reported a significantly better overall trial experience compared to controls (experience in intervention arm: median 60, IQR 54-63; experience in control arm: median 55, IQR 40-62; *P*=.01). In the intervention arm, N=45 80% created an account, with a median of 4 (IQR 2-6) sessions and total engagement length of 7.6 (IQR 2.2-15.1) minutes of total engagement. Figure 2 indicates that most participant engagement with the app was concentrated at the beginning of their invitation to the trial and tailed off an average of 4 weeks after randomization. The app did not include reminders or notifications aside from 6-week and 12-week surveys. Content was comprehensive across mental, physical, social, and real-world health during the postpartum period, and use was captured through Amplitude. There were 2 notable findings from content analytics. Participant bookmarked content that ranged across all areas (N=25% mental, N=36% physical, and N=39% social and real-world). PPD and mastitis were the most commonly bookmarked red flags.

### Depression Scores and Frequency

Overall depression scores from the EPDS averaged 9.5, with intervention scores averaging 9.7 and control group scores averaging 9.3. In the overall sample, 60 (45.5%) of the 132 analyzable participants reported a summary score of >11 or a positive response on question 10 (self-harm), which was defined as at risk for depression. As part of our safety monitoring protocol and to address the concern that the birthing parent can be “forgotten” during the postpartum period, all participants identified as being at risk were contacted to ensure they were aware of their risk score and to inquire about resources and access to support.

### Qualitative Outcomes of Open-Ended Responses

Across all open-ended responses to “What postpartum-related apps do you like?” and “Please select the platforms you are most likely to use for communicating with others,” the following five interconnected themes emerged: (1) Limited Awareness and Use of Postpartum Apps: most participants reported little to no use of postpartum-specific apps, instead citing general wellness or pregnancy tools such as Flo, Glow Baby, Peanut, and What to Expect. This indicates a broad gap in awareness, accessibility, and availability of digital tools tailored specifically to the postpartum experience. (2) Reliance on Peer and Social Platforms: participants described frequent engagement with peer-to-peer online communities (eg, Facebook, Instagram [Meta Platforms, Inc], Discord [Discord Inc], and Reddit [Reddit, Inc]) for advice and emotional support. These social platforms were valued for relatability and a sense of belonging, underscoring that many women seek connection and shared experience rather than purely informational content. (3) Preference for Simple, Direct Communication: When asked about preferred communication channels, text messaging and phone calls were most commonly selected, followed by social media and email. Participants favored low-effort, familiar methods over downloading new apps, suggesting that convenience and immediacy are critical for engagement during the postpartum period. (4) Limited Mentions of Specialized Tools: only a few respondents referenced niche postpartum or tracking apps (eg, Ovia, LactApp, and Baby Tracker). These were used primarily for task-based functions such as logging feedings or sleep, with minimal emphasis on mental health or well-being content. This may highlight an unmet need for broader postpartum tools. (5) Central Role of Community and Emotional Support: across both questions, mothers emphasized the need for empathy, shared understanding, and emotional validation. Rather than seeking static information, they expressed a desire for authentic support from peers and trusted sources.

### Predictive Model Outcomes

The predictive model demonstrated strong predictive performance for identifying women at risk of PPD based on clinical trial data (defined as having an EPDS score ≥10). On the held-out test set (N=24), the optimized model achieved a recall of 0.89 and a precision of 0.73. Of all participants truly at risk (EPDS ≥10), 89% were correctly identified by the model, while of all participants predicted as “at risk,” 73% were actually at risk.

Feature importance analysis identified key predictive variables for early screening. The baseline STAI score was the strongest predictor of PPD risk. This finding aligns with the exploratory data analysis, which showed a high correlation between STAI and EPDS scores. The next four most important features were CDRS_baseline, CDI_baseline, MOS_baseline, and income between US $20,000 and US $39,999.

It is important to note that this model was trained on a relatively small dataset (N=119, with n=24 in the test set); therefore, the results should be interpreted as a proof-of-concept rather than a validated predictive model. While these performance metrics provide a strong signal of predictive strength and demonstrate the feasibility of this modeling approach, the estimates would be more stable and reliable with a larger dataset.

All analyses were conducted using deidentified data under the requirements of Good Clinical Practice and the WIRB-Copernicus Group Institutional Review Board.

### Platform Experience

Descriptive experience data demonstrated that participants somewhat agreed, agreed, or strongly agreed that the platform was evidence-based N=50 (89.3%); the content was easy to understand N=52 (92.9%); and useful N-49 (87.5%). Additionally, participants reported that the platform was actionable N=49 (87.5%), easy to use N=52 (92.9%), delivered at the right length N=51 (91.1%), and relatable N=52 (92.9%). While N=36 64.3% of users did not access red flags, and N=20 35.7% did engage with this feature. Additionally, N=22 39.3% reported that they followed up with a care provider or sought emergency care based on the information Joyuus, and N=28 50% followed up on resources based on the information presented in Joyuus.

## Discussion

### Principal Findings

The trial demonstrated the feasibility and acceptability of the Joyuus platform, even though improvements in measured outcomes were not statistically significant. The absence of statistically significant differences between groups suggests that the current intervention design may be insufficient to drive measurable change in outcomes over 12 weeks. Engagement decline over the first 4 weeks mirrors common challenges in postpartum digital interventions and indicates that sustained interactions may require additional strategies, such as enhanced onboarding, adaptive content delivery, peer connection, targeted prompts, and clinician integration, which could improve outcomes. These are all features in development based on the study findings. Future iterations of the platform will build on these findings to incorporate strategies and mechanisms to improve sustained engagement.

The difference in trial experience between intervention and control groups underscores that Joyuus provided perceived value and emotional support, despite limited change in scores between timepoints. These findings suggest opportunities to improve engagement. While there are limited studies on measurement reactivity, it is a possible explanation for intervention and control score differences between baseline and 12 weeks. The act of completing assessments, along with content that raises awareness of depressive symptoms, might itself influence participants’ self‐reporting of symptoms at the 12-week timepoint. Additionally, important information was gained regarding the time period during which individuals are most likely to engage without incentives. It was intentional and important to develop a program for use in the real world, and this included allowing individuals to use the platform when and how they needed, rather than including artificially incentivized reminders to log in. While this may have contributed to the lack of significant change between timepoints, it created a window into the need for such a program and actions to improve value to the end-users. This informs how we approach critical actions, such as determining at-risk populations during onboarding and within the first few weeks of signing up to the platform. These data also provide potential for additional forms of engagement, based on the quantitative and qualitative data. The open-ended qualitative findings indicate that individuals are most in need of broad information, yet for specific and tangible questions, suggesting that continuing to develop our responsive approach to delivering information can create a more tailored experience, making information more easily accessible and actionable. Finally, the engagement data provide insights into what types of engagement are important, including the need for a specific and comprehensive postpartum platform, connection to existing postpartum communities, and direct and clear information and connection to actions.

An important outcome for our initial use case of depression was revealed during this study. Joyuus identified risk in postpartum individuals in the intervention and control arms. Identification of higher-than-average depression rates (44%) and depression risk scores (9.5) underscores the fact that individuals continue to be underdiagnosed despite higher prevalence rates than typically reported. These rates ranged across a broadly diverse demographic sample.

Further, our predictive data modeling demonstrates that when demographic factors and indicators of risk for depression are captured, Joyuus is strongly predictive of depression (0.89 recall) without the requirement of completing the EPDS. While the EPDS is the gold standard for determining risk, our goal was to demonstrate that the indication of risk can be addressed earlier and that alerting individuals to the potential risk can lead to seeking services before risk becomes more severe. In addition, Joyuus includes the complete EPDS scale as part of the platform, and individuals who are identified as potentially at risk can complete the EPDS and share those findings with their care provider. Future iterations of Joyuus are exploring personalized engagement and adaptive algorithms to sustain participation and target at-risk subgroups.

Despite the results related to our main outcomes, it is important to recognize that even if a subset of intended participants adopts and benefits from the platform, there is a meaningful potential for significant change in health outcomes. For instance, although use occurred primarily in the first 4 weeks after randomization, we noted that participants in the intervention group reported that the content was of interest, and depression content was one of the top bookmarked topics. Data also showed that individuals who engaged with the program in the intervention group reported that they would seek additional information and found the program relevant, evidence-based, and actionable.

### Limitations

A few challenges and limitations of this study must be noted. Our study was conducted entirely online to capture a national sample during the first 6 months postpartum. Online-based research faces growing challenges in ensuring that the identified sample is actually being recruited. While our approach was to work directly through community-based groups of postpartum individuals, the post was broadly shared at 2 different timepoints, resulting in bot, fictitious, duplicate, or otherwise fraudulent entries. Because these invalid submissions were identified and removed before baseline enrollment through active monitoring and verification procedures, the total number of initial screening attempts could not be reliably quantified. These data were identified through both Qualtrics standard bot and duplication detection, and research associates monitored latitude and longitude data to identify the country of origin or concentration of would-be participants. We found that, in both instances where we saw a significant increase in numbers, data were being received from outside of the United States or were heavily concentrated in very specific areas within cities such as Atlanta, Georgia, or Los Angeles, California. These events triggered verbal (phone or Zoom [Zoom Communications, Inc]) contact to confirm that participants met the eligibility requirements and were not bots. Based on these interactions, we created a new anonymous survey link and reached out through new community partners who had direct contact with the potential participants. The analyses and recruitment metrics are reported based on validated participants who met eligibility and completed baseline enrollment. We intentionally did not set the platform up with significant reminders, either through notifications or research contacts. Notifications are increasingly becoming considered a nuisance to end users, and research-driven contact sets false expectations of the effectiveness of the intervention. It is likely that without notifications of any kind, busy individuals were initially engaged, received the information of interest, and lost track of the app over time. These data have been valuable in supporting efforts to develop appropriately targeted engagement activities.

### Conclusion

This study provides early evidence that a comprehensive tool for postpartum support fills an existing gap in the postpartum care continuum. While user participation dropped off after the initial month of use, user feedback suggested value in several components. Our study demonstrates that a platform intended to engage across a range of postpartum content and conditions can engage individuals where and when they are looking for care, with the potential to identify and predict risks for disease before they increase in severity. These findings suggest that postpartum women prioritize connection, simplicity, and trust over technology complexity. Digital postpartum platforms integrating clinical and nonclinical factors may support earlier identification of risk, with further research to determine their impact on clinical outcomes and care integration. Increased knowledge of risks has the potential to improve outcomes with increased engagement and connection to care through partnering with community, safety net, and health benefits providers. Pilot studies to test this model are planned to fully assess the impact on outcomes and quality of care.

## Supplementary material

10.2196/89719 Checklist 1 CONSORT-eHEALTH (V 1.6.1) checklist.

## Abbreviations

BIMF: Barkin Index of Maternal Functioning
CD-RISC: Connor-Davidson Resilience Scale
CONSORT: Consolidated Standards of Reporting Trials
EPDS: Edinburgh Postnatal Depression Scale
MOS: Medical Outcomes Study
OHRP: Office for Human Research Protections
PPD: postpartum depression
STAI: State-Trait Anxiety Inventory
